# Toward the effective and fair funding of CO_2_ removal technologies

**DOI:** 10.1038/s41467-023-36199-4

**Published:** 2023-02-07

**Authors:** Matthias Honegger

**Affiliations:** Perspectives Climate Research gGmbH, Freiburg i.B., Germany

**Keywords:** Technology, Climate-change mitigation, Climate-change mitigation

## Abstract

Carbon dioxide removal technologies are gaining prominence in academia, industry and policy, yet the need for substantial funding raises serious challenges. This comment discusses these issues and offers suggestions for future funding efforts in this area.

## Teaser

Carbon dioxide removal technologies are gaining prominence in academia, industry and policy, yet the need for substantial funding raises serious challenges. This comment outlines these issues and charts a path for the effective, systematic and fair mobilization of funds for removals.

## CO_2_ removal and geological storage necessary for net-zero

Increasingly, social scientists, philosophers and climate scientists agree that CO_2_ removal technologies are necessary in tandem with decarbonization^1^. Mainstream scenarios foresee removal of several billion tCO_2_ per year, as well as additional storage to properly decarbonize industry^2^. Various technological methods for removing CO_2_ are in development, primarily capturing CO_2_ directly from the air (direct air carbon capture and storage (DACCS) or through biomass processing and storage. While DACCS represents an industrial activity, biomass-based removal can—and arguably should—take place in all biomass-processing sectors; waste, energy, and productive industries such as cement, steel and chemicals. The supply of sustainable biomass feedstock through agricultural and forestry activity is also a crucial part of the equation. Despite developments in this area, current carbon capture and storage (CCS) projects in the early planning stage yield a storage capacity of just 0.2% of global annual emissions^2,3^, and underground carbon storage facilities need to be rapidly scaled up.

Despite the IPCC stating that CO_2_ removals are necessary for decreasing net emissions, as well as achieving net-zero and later net-negative emissions^2^, policies are largely missing. Ethicists, social scientists, and environmental advocacy organizations have concerns that directing funding towards uncertain future technologies may undermine current mitigation efforts^4^, while activists fear that removals could extend a lifeline to the fossil fuel sector. Funding has thus to date been inadequate for the multi-billion-ton scale of CO_2_ removal needed^5^. While some governments have supported individual geological CO_2_ storage sites, these are insufficient for meeting existing obligations under the Paris Agreement. Governments across the globe will therefore need to mobilize funding in line with their net-zero ambitions. Doing this equitably means that historical emitters need to be among the first movers. I argue that more political accountability is needed: a coalition of countries should step forward at the 2023 UN Climate Change Conference (COP28) and commit to scaling funding with intermediate net-zero aligned milestones. The coalition should also facilitate peer-learning as an incentive to attract less technologically advanced countries.

## Current efforts to fund CO_2_ removal

The technology to capture CO_2_ at specific localized sources is mature and applicable to waste-incineration plants, thermal power plants, cement, or other industrial plants. Where such processes involve biomass, they contribute to removing CO_2_ from the atmosphere. The IEA^6^ estimates costs of point-source CO_2_ capture (15–120$/tCO_2_), transport (2-14$/tCO_2_) and storage (−23-55$/tCO_2_). Negative costs are associated with using CO_2_ for enhanced oil or gas recovery. Although significant cost reductions are expected in the future, direct capture of CO_2_ is costlier—at nearly 1000$/tCO_2_—due to the lower CO_2_ concentration.

Governments have yet to take responsibility for funding CO_2_ removal at scale. To date, most funding for CO_2_ removal has been voluntary and via the private sector including encouraging investments in innovation. For example, Frontier, from payment service provider Stripe, bundles funding for investment from thousands of companies using “stripe climate”, which is a transaction-fee based donation option. The Climate Transformation Fund—a cooperation between the Milkywire climate fund and payment service provider Klarna, channels investments in a similar manner. Microsoft is also a notable buyer of CO_2_ removal certificates, although its priority of compensating present-day emissions has led to also funding mature technologies and low-cost forestry projects. Voluntary carbon market standards have also started to adopt technological removals, such as the Verified Carbon Standard (VCS) adopting a range of new project types in 2023. These will allow a much larger range of companies and individuals to become buyers of removal certificates in the future. While these can be important pioneers, voluntary markets have historically remained orders of magnitude smaller than compliance markets induced through public policies^7^. Relying exclusively on voluntary action in the long-term could be likened to relying on donations to fund public sewage systems.

A range of experimental national policies are emerging. For example, Sweden is pursuing reverse auctions to allocate public service contracts that deliver biomass energy with carbon capture and storage efficiently^8^. Switzerland has crafted an agreement with its waste-incineration sector to implement at least one significant facility with carbon capture for storage abroad by 2030^9^. Additionally, the United States in August 2022 adopted a historic climate mitigation investment package as part of its Inflation Reduction Act, which promises an unprecedented volume of funding for CO_2_ removal including through its tax credit 45Q^10^. While a promising start, national-level efforts need to be strengthened and normalized internationally to mobilize CO_2_ removal technologies at rates commensurate with 2050 net-zero targets^11^. Although governments have tacitly committed this in their Paris Agreement contributions and net-zero pledges, they have to date largely shied away from triggering the necessary investments.

## Policy tools for incentivizing CO_2_ removals and sharing the costs

The ad hoc nature of efforts to date raises important questions for the future of equitable financing in this area. Given the cost and scale of the issue, CO_2_ removal for keeping to a well-below 2 °C pathway could require mobilization of billions of dollars annually. Various approaches to mobilizing funds can be imagined, each of which has its strengths and weaknesses as well as country-specific opportunities and challenges.

The “polluter pays” principle may seem appropriate. However, it is politically complicated, as imposing costs on industry or energy producers could cause cost to pass-through to consumers and induce political counter campaigns and public backlash. In the past, some fossil fuel or power companies have raised gas or power prices and successfully blamed climate policies. The option of mandating entire sectors to pursue both CO_2_ capture for emissions reductions and CO_2_ removal should nonetheless be on the table. Such regulation would impose cost absorption, although sectors may differ in their ability to absorb costs given variation in exposure to international competition and margins^12^. Some sectors, such as waste treatment and cement, have a captive clientele due to immobile products or services, and may more easily internalize decarbonization and CO_2_ removal costs. More exposed sectors (such as chemical, pulp and paper and steel industries) may require more protection or support. However, no sector—even if considered “hard to abate”, such as cement, aviation and agriculture—should be exempt. Tailored incentivisation across sectors and countries is needed for an adequate portfolio of CO_2_ removal options to emerge.

As subsidies may be constrained through public budgets, carbon markets could help gradually mobilize private sector funding. Pure subsidies may from the outset incorporate a phase-out period, to avoid public backlash and prevent inefficiencies. Additionally, tax breaks could be suitable instruments, especially where fossil fuel-producing states can give up associated tax revenue. However, proceeding with subsidies and tax breaks requires caution to avoid pressure on lower-income households through inflation or other indirect effects.

There is no one-size fits all approach to policy design hence why the Paris Agreement is premised on national policy planning. Committed governments (especially historical emitters) must now take action through policies that work in their national political systems to scale removals and emissions-reducing CCS in line with respective mitigation targets.

## A carbon management commitment coalition

I call for a coalition of climate leaders – including historical emitter countries – to step forward and publicly commit to executing necessary policy steps in line with existing net-zero targets at COP28. Ideally, coalition members would declare quantitative milestones for the scaling of both removals and CO_2_ storage aligned with their respective net-zero target years. They would each commit to devising a nationally best suited approach to funding CO_2_ removals, drawing on advantages each of the above policy tools have to offer and expanding on existing instruments where appropriate. In industry sectors, where feasibility has been established, governments could impose removals-obligations. These could require relevant sectors (e.g., waste, industry and energy) to scale removals either through sector agreements or market-based instruments that mandate a rising per cent-share of removals. To mobilize efficient action across national borders, the coalition should also pledge international cooperation through carbon markets. Markets, along with technology transfer and climate finance should help unlock potentials in the Global South^1^ and ensure that more countries join the coalition.

High standards and transparency in planning and monitoring will be key – including for differentiating CO_2_ removal and emissions reductions, both of which need to be incentivized adequately to meet net-zero targets. Conversely, zero-emissions power, sustainable biomass, and water may become critical, competing factors in net-zero strategies as they are shared requirements for emissions-reducing and GHG removal technologies. Coalition members should thus pledge to prevent potential resource competition within mitigation portfolios to avoid funding an ensemble of technologies that cannot scale.

Mobilizing CO_2_ removal and associated funding effectively, systematically, and fairly may seem daunting and will require critical, constructive voices and regular course corrections. However, a well-orchestrated coalition effort could unlock a step-change and mobilize decisive policies and funding to scale CO_2_ removals and storage.
